# Impacts of a Standing Desk Intervention within an English Primary School Classroom: A Pilot Controlled Trial

**DOI:** 10.3390/ijerph17197048

**Published:** 2020-09-26

**Authors:** Aron P. Sherry, Natalie Pearson, Nicola D. Ridgers, William Johnson, Sally E. Barber, Daniel D. Bingham, Liana C. Nagy, Stacy A. Clemes

**Affiliations:** 1 National Centre for Sport and Exercise Medicine, School of Sport, Exercise and Health Sciences, Loughborough University, Loughborough LE11 3TU, UK; N.L.Pearson@lboro.ac.uk (N.P.); W.O.Johnson@lboro.ac.uk (W.J.); S.A.Clemes@lboro.ac.uk (S.A.C.); 2 NIHR Leicester Biomedical Research Centre—Lifestyle Theme, Loughborough University, Loughborough LE11 3TU, UK; 3 Institute for Physical Activity and Nutrition (IPAN), School of Exercise and Nutrition Sciences, Deakin University, Geelong 3220, Australia; nicky.ridgers@deakin.edu.au; 4 Bradford Institute for Health Research, Bradford Teaching Hospitals Foundation Trust, Bradford BD9 6RJ, UK; Sally.Barber@bthft.nhs.uk (S.E.B.); Daniel.Bingham@bthft.nhs.uk (D.D.B.); 5 Centre for Applied Education Research, Wolfson Centre for Applied Health Research, Bradford Teaching Hospitals Foundation Trust, Bradford BD9 6RJ, UK; 6 Faculty of Health and Life Sciences, Oxford Brookes University, Oxford OX3 0BP, UK; lnagy@brookes.ac.uk

**Keywords:** sitting time, standing desks, sit–stand desk, children, primary school, classroom interventions, physical activity

## Abstract

Traditional classroom furniture dictates that children predominantly sit during class time. This study evaluated the impact of providing standing desks within a deprived UK primary school setting over 8 months using mixed-method approaches. All children within a Year 5 class (9–10-year-olds, *n* = 30) received an adjustable sit–stand desk, while another Year 5 class (*n* = 30) in a nearby school retained traditional furniture as a control classroom. At baseline, 4 months, and 8 months, activPAL monitors (PAL Technologies, Glasgow, UK) were worn for 7 days to provide time spent sitting and standing. Behavior-related mental health, musculoskeletal discomfort surveys, and a cognitive function test battery were also completed at all three timepoints. Intervention experiences from pupils and the teacher were captured using focus groups, interviews, and classroom observations. At both 4 months and 8 months, multi-level models revealed a reduction in class time sitting in the intervention group compared to the control group ((β (95%CI) 4 months −25.3% (−32.3, −18.4); 8 months −19.9% (−27.05, −12.9)). Qualitative data revealed challenges to teaching practicalities and a gradual decline in behavior-related mental health was observed (intervention vs. control: 4 months +5.31 (+2.55, +8.08); 8 months +7.92 (+5.18, +10.66)). Larger trials within similar high-priority settings are required to determine the feasibility and cost-effectiveness of providing standing desks to every child in the classroom.

## 1. Introduction

Sedentary behavior is defined as “any waking behavior characterized by an energy expenditure ≤ 1.5 METs while in a sitting, reclining or lying posture” [1]. Recent inclinometer data from a sample of children from a deprived setting in the UK has shown that they spend 10–11 h/day (68–74% of wear time) sitting on weekdays and weekend days [2]. Such excessive daily sitting has been reported to become more detrimental to morbidity and mortality outcomes as a person transitions through the life-course [3,4,5]. Sitting during childhood tracks into adolescence and adulthood [6,7], with time spent sitting increasing during these transitions, particularly between childhood and adolescence [8]. Consequently, interventions to reduce sitting time during late childhood could be pivotal in curtailing current sitting behavior trends. There is clear evidence that investment into children’s health can provide longer-term benefits to health and development [9].

Given children spend half of their waking hours at school, the school environment may provide a critical influence on their health behaviors [10]. The classroom environment is traditionally associated with high volumes of sitting. A recent study demonstrated that children and adolescents (8–15 years) were sedentary/stationary for 70–90% of the time during different school subjects [11]. It may therefore be opportune to target the classroom setting, a highly structured and controlled environment, to reduce sitting across a large population of children. This could be achieved by restructuring the physical classroom environment without adding to often crowded school curriculums [12].

The use of standing desks has received increasing attention within primary/elementary school classrooms in recent years [12,13] as a promising solution for reducing total sedentary time [14,15,16]. The simple self-service design (the user can freely transition between sitting and standing) of adjustable sit–stand desks (a type of standing desk) is a major strength compared to other common strategies (i.e., educational classes on the benefits of reducing classroom sitting) that may have a greater dependence on teachers [12]. The autonomous, subconscious nature of using standing desks may also play a part in their relative success [15]. The user has the ability to not only reduce total sitting time but frequently interrupt prolonged bouts of sitting with standing or light physical activity.

Standing desk studies within the primary school classroom to date have mostly been limited to relatively short (<4 weeks) to mid-length (<6 months) intervention durations, with contrasting intervention approaches and study designs [13,17]. Studies have demonstrated reductions in time spent sitting in class and increases in standing or stepping time [18,19,20,21,22,23]. Intervention effects on health outcomes such as body mass index (BMI) [21,23,24], waist circumference, blood pressure [22] and musculoskeletal discomfort have been mixed [22,25,26], but consistent increases in energy expenditure have been observed [27,28,29].

Although health outcomes in children are important, it is critical that the impact of standing desks on aspects of classroom behavior, learning and development-related outcomes (including cognitive function, for example) are explored at such a key stage of life [16,30]. Very few standing desk studies have explored the impacts on these outcomes in children to date [19,21,31]. These outcomes will be a priority for parents and teachers when considering the longer-term adoption of this intervention approach, and require further attention. The limited evidence suggests that standing desks are not detrimental to development or learning [19,21,31]; however, these studies were 5 months in duration and the longer-term impacts are unknown. The absence of negative effects on development-related outcomes will need to be replicated in longer-term studies if standing desks are to be accepted as a permanent classroom fixture within an educational setting [12].

An important benefit of classroom-based interventions is that they are accessible to all children that attend school, offering a way to reduce health inequalities. Children of low socio-economic position experience a wide range of adverse health and development outcomes [32,33] and are more likely to develop a range of chronic diseases in adulthood compared to peers [34]. Ethnic minority children are overrepresented in deprived neighborhoods in the UK [35] and are more likely to be sedentary at and away from school and perform less moderate to vigorous physical activity [36,37,38]. South Asians (the second most common ethnic group in the UK [39]) have demonstrated an increased risk of type 2 diabetes and cardiovascular disease compared to White Europeans in childhood [40,41]. However, to date, just two standing desk studies [18,23] have been implemented in the UK within such higher-risk populations

School-based standing desk studies have almost exclusively implemented a partial desk allocation system where several standing desks replace a few traditional desks and children are rotated [12,13,42]. Although this approach will be more financially viable than a full desk allocation system (a standing desk for every child), intervention exposure time will be substantially reduced. Opportunities to be active when located at a standing desk (i.e., standing, light ambulation) are likely to be of low intensity although it can provide meaningful contributions to total daily physical activity [43]. Greater standing desk exposure time from a full desk allocation system would maximize the opportunities to replace sitting with physical activity compared to a partial desk allocation system. Consequently, potential health benefits yielded from standing desks may be optimal when children have maximum exposure. Only one study has implemented a full desk allocation system within a primary school classroom, located in Australia [18,22]. The impacts of implementing a full desk allocation system within UK primary schools on sitting behavior, physical activity, and health and development is unknown; such information is important for shaping full-scale randomized controlled-trials where intervention effectiveness can be evaluated.

The primary purpose of this study therefore was to assess the impact of a full standing desk allocation system on sitting behavior over an 8-month period in a Year 5 (9–10-year-olds) UK primary school classroom within a relatively deprived location. The secondary purpose was to explore changes in behavior-related mental health, musculoskeletal health, and markers of cognitive function. Finally, child and teacher attitudes, experiences and behaviors when implementing and using the intervention were explored using focus groups, interviews, and direct observations.

## 2. Materials and Methods

### 2.1. Study Design and Setting

This pilot controlled trial was implemented within two primary schools with similar characteristics (i.e., two or three classes per year, seven year groups in total, children aged 4–11 years) in the city of Bradford, UK. Separate schools were used to avoid contamination between control and intervention classrooms [18,22]. The two schools were located within 3 km of each other, within deprived neighborhoods (top 30% in the UK [44]) and had similar class sizes (*n* = 30 approximately). These schools were invited to participate due to their engagement with the Born in Bradford Project [45], a large-scale longitudinal health research project, which has connections to local schools.

### 2.2. Participant Recruitment and Ethical Approval

Year 5 children (aged 9–10 years) were targeted for this study due to their more active participation in learning [46], cognitive ability to complete study measures and to expand on a previous pilot study [18]. Furthermore, large increases in the time spent sedentary (in total and in prolonged bouts) from childhood into adolescence occur between the ages of 9 and 12 years of age [8], making this age group an important target for interventions.

Classes to be involved in the study were selected by school management after the class teachers were consulted. Parents/guardians of children within these Year 5 classes received a letter sent via the school detailing the study along with an opt-in consent form for their child to participate in the evaluation measures. Children without parental consent were still exposed to the standing desks in their classroom if happy to use one. Children with parental consent were required to provide verbal and written assent prior to any evaluation measurements being taken. The study was approved by Loughborough University Ethical Advisory Committee (project ID: R15-P086). The study has clinical trial registration (NCT04296669, registered 4 March 2020). Children were excluded from the evaluation measures if they had a disability that prevented periods of standing or an injury or illness that precluded the ability to perform normal daily tasks.

### 2.3. Intervention

One school was allocated to receive the intervention by the research team at the study planning stages. Within this school, all pupils within a single class received an Ergotron LearnFit height adjustable sit–stand desk that allows the user to manually shift between sitting and standing. These desks have been used in recent school-based standing desk studies [22,23,47] The sit–stand desks were installed in the intervention classroom two weeks after baseline measurements (November 2015) and traditional classroom stools were retained for use with the new desks. Before the intervention began, pupils and teaching staff were trained by research team members in how to position the desk to the correct height while sitting and standing according to the manufacturer guidelines. Instructional posters were also positioned around the classroom demonstrating correct posture. The intervention school timetable was 08:50–15:10 with 60 min in total for break times, providing a maximum classroom time and sit–stand desk exposure of 5 h 30 min.

The intervention was designed using the Behavior Change Wheel (BCW) [48]. The targeted behavior was class time sitting. A mediating process of children reducing sitting time in the intervention group was identified as children choosing to stand rather than sit during class time and the standing desks were identified as a direct influence on the mediator. The standing desks were identified as an enabling factor for changing class time sitting via physical opportunity within the COM-B model. To demonstrate standing in class as a social norm and therefore provide social opportunity (COM-B model), a daily standing class was agreed with the lead teacher where all children were instructed to stand for the first 20 min of a mathematics class. This class was chosen by the teacher as it occurred in the morning daily. Reflective motivation (COM-B model) of the teacher was identified as a key variable for sufficient intervention implementation and adherence. This was targeted using a Professional Development Manual, adapted from a resource used in a previous study [18], and from the lead researcher visiting the school on a monthly basis to provide in-person support and re-enforce the importance of the intervention and the teachers’ role within it. The Professional Development Manual also included instructions on how to lead a standing class and correct postures when sitting or standing at the desks.

### 2.4. Control Group

Within the other school, a single class functioned as a control group, continuing with traditional classroom furniture and normal lessons. The control school timetable was 08:40–15:15, with 60 min break time, providing a maximum classroom time of 5 h 45 min

### 2.5. Measures

Baseline measures began in autumn (November) 2015 and the study concluded in summer (July) 2016. There were three measurement points during the study; baseline, 4 months (mid-intervention; February 2016) and 8 months (children continued to have access to the intervention during the final follow up). The same measures were conducted at each time-point by trained research staff in both the intervention and control schools. At baseline, children self-reported their age and ethnicity (after ethnicity was explained and a subsequent selection was made from a list of options i.e., white British, Murpuri Pakistani).

#### 2.5.1. Quantitative Measures

##### Sitting and Physical Activity Behavior

All participants wore an activPAL inclinometer (PAL Technologies Ltd., Glasgow, UK) on the anterior aspect of the right thigh, placed within a nitrile sleeve and attached using hypoallergenic medical dressing (Hypafix, BSN Medical, Hull, UK), for 7 days. Tri-axial data were sampled at 20 Hz. The device was waterproofed (using Hypafix and the nitrile sleeve), enabling a 24 h wear protocol. Sitting data were explored during class time (based on timetables), after school, and during total waking hours on weekdays. Participants were included in the analyses if they provided ≥8 h of activPAL data per day [22] on ≥2 weekdays [18,23]. Sleep time (11pm–6am) was removed from the data [49]. A non-wear time of 20 min was also applied using the accelerometer function, determining additional sleep periods (between 6am and 11pm) or non-wear periods during waking hours [49,50]. The non-wear time and epoch parameters are consistent with previous activPAL research [49], and are recommended in children [50]. activPAL data were downloaded using standard manufacturer software (activPAL Professional v.7.2.32, PAL Technologies, Glasgow, UK). Files were converted to 15-s epochs and then processed with a customized Microsoft Excel macro [18]. The customized macro provided the frequency of and accumulated minutes spent sitting, standing, and stepping and sit-to-stand transitions. Excel macro data were subsequently cleaned in Stata (StataCorp LP., College station, TX, USA) and the proportion of wear time spent sitting or in different modes of physical activity were then calculated [18].

##### Anthropometrics

Anthropometric data were collected for descriptive purposes. Height (portable stadiometer: Seca UK, Birmingham, UK) and weight (portable electronic weighing scales: Seca model 887) were recorded to the nearest 0.1 cm and 0.1 kg, respectively, with shoes removed. BMI was calculated (weight (kg)/height (m)^2^) and z-scores and percentiles determined using the British 1990 growth references [51]. Percentiles were then used to allocate individuals into either an underweight, normal, overweight or obese category [51].

##### Behavior-Related Mental Health

The 25-item Strength and Difficulties questionnaire [52] was completed by teachers to assess five scales of mental health; emotional problems, conduct problems, hyperactivity, peer problems, and prosocial behavior for each child. Each item includes a statement which is responded to by either selecting “not true”, “somewhat true”, or “certainly true,” which are coded as 0, 1, and 2 respectively for all but five random items, which are reverse coded. Scores are totaled for each scale and overall (excluding the prosocial scale) and each score is categorized using standardized cut points based on a UK community cohort of 4–17-year-olds [52]. These categories include “close to average” (0–13), “slightly raised” (14–16), “high” (17–19), and “very high” (20–40) risk of a behavioral disorder. This questionnaire, when completed by teachers, has been shown to be a valid measure of children’s behavior (convergent validity: Pearson correlation coefficient with the Rutter questionnaire = 0.92) [52].

##### Musculoskeletal Discomfort

Musculoskeletal discomfort was measured using a seven-item survey, comprising different body parts (neck, arm, back, wrists/hands, hips, legs, ankles/feet) on a 5-point scale, with ratings ranging from “good” to “OK” to “bad” in terms of comfort (later coded 1–5 (good to bad) for each body part). All seven scales were combined to produce an overall mean discomfort score. This survey has been used in a previous classroom-based standing desk study [53].

##### Cognitive Function

Cognitive function was assessed using a battery of two computer-based tests: the Stroop test and the Corsi Block Tapping test. The Stroop test assesses executive function where participants must correctly select the font color of a target word, ignoring the actual target color spelled out [54]. Reaction time was the key outcome, with the mean baseline reaction time subtracted from the interference reaction time to determine sensitivity to interference. The Corsi Block Tapping test measures visual spatial working memory capacity [55]. Participants are presented with a 3 × 3 grid of squares and a sequence occurs by individual squares temporarily changing color in which the participant must accurately repeat. The sequence increases or decreases by 1 with every correctly or incorrectly repeated sequence respectively, with a minimum (and starting sequence) length of 3 and maximum of 12. The key outcome is mean sequence length across 12 attempts. The battery was performed on each child’s own school laptop without touch screen application, taking approximately 10 min to finish but at each participant’s own pace. The battery was completed once per measurement point following a familiarization attempt the previous day. These tests have been used in physical activity-related research with children previously [56,57].

#### 2.5.2. Qualitative Measures

##### Interviews

A brief semi-structured interview was conducted with the teacher of the intervention group and focus groups were conducted with six randomly selected intervention pupils (two groups of three), nine weeks after standing desk installation, to avoid impacting on primary and secondary outcome assessments. The interview questions were developed based on a list of factors that can influence the implementation process [58]. The planned focus group and interview questions, and how the questions were generated, are provided as Supplementary Materials (Supplementary File S1). Child and teacher responses to interview/focus group questions were handwritten by the lead researcher.

##### Classroom Observations

Two 30-minute classroom observations were conducted within the intervention class by research team members, who recorded field notes based on these observations [59], on day one and during week 16 of the intervention. The first observation took place during a normal-practice morning class and the second during a teacher-instructed standing class. The standing classes were agreed between the teacher and research team to take place every day during mathematics. During this observation, the researcher made field notes on the children’s responses (e.g., positive or negative responses) to the enforced standing class, the children’s perceived attitude towards standing during this class, and sitting and standing behavior immediately after the enforced standing class. A negative response or attitude was identified from signs of disappointment or reluctance to stand (e.g., groans/complaints), whereas positive response were interpreted as an absence of negative responses or enthusiasm/excitement to stand (e.g., smiling, positive comments).

### 2.6. Statistical Analysis

Statistical analyses of quantitative data (activPAL, behavior-related mental health, cognitive function, musculoskeletal discomfort) were conducted using Stata 15.0 (StataCorp LP., College station, TX, USA). Baseline comparisons were made between the control group and intervention group across outcome variables. Categorical data (sex, BMI categories, ethnicity) were compared using Pearson chi-square tests. Continuous data (all other variables) were checked for normality within baseline using Kolmogorov–Smirnov tests prior to baseline comparisons. The Kolmogorov–Smirnov test confirmed both normally distributed and skewed data. Normally distributed data sets were compared between groups using independent *t*-tests. For skewed data, a natural log transformation was applied. Transformed data were then compared between groups using independent *t*-tests. Mean transformed values and confidence intervals were then back transformed and reported in the results. Data that were still skewed following transformations were compared between the intervention and control group using the Wilcoxon signed-rank test, and the median and interquartile range reported. To account for differences in wear time in activPAL data, the proportion of wear time spent in different activities (i.e., sitting, standing, stepping) were included in the subsequent analysis. Multi-level modelling was applied to determine the influence of the intervention on sitting and physical activity, behavior-related mental health, cognitive function, and musculoskeletal discomfort (see Supplementary File S2). All models were univariate which were fitted to all outcome (dependent) variables of interest. The data were structured as occasions (level 1) nested within individuals (level 2).

Qualitative data (focus groups with children, interview with the teacher, direct classroom observations) explored experiences and perceptions of the standing desk intervention. Due to the limited time available when conducting the interviews and focus groups, a small sample of planned questions with both the pupils and the teacher were asked, resulting in a low volume of raw data being collected. General key themes from the data were therefore summarized within the results rather than using any analytical procedure.

## 3. Results

### 3.1. Descriptive Characteristics

Fifty-five children across the two schools had parental consent and assented to take part in the study (28/31 control group, 27/30 intervention group, 90% overall). Fifty-three participants (96% of those with parental consent) provided valid activPAL data at baseline and were subsequently included in the study for all quantitative and qualitative analyses. Sample characteristics are reported in Table 1. At baseline there was a significant difference between classes in the proportion of South Asian and White British children and in activPAL wear time during class time and a full day (Table 1). No other differences were observed at baseline. Completion rates for quantitative outcome measures for both groups at baseline, 4 months, and 8 months are presented in Table 2. See Supplementary Table S1 for activPAL descriptive data.

### 3.2. Quantitative Data

#### 3.2.1. Sitting and Physical Activity

Table 3 shows the estimated effect sizes of the intervention on activPAL-determined sitting, standing, and stepping during class, after school and throughout waking hours on weekdays at baseline, 4 and 8 months, from the multi-level models. Compared to the control group, the intervention group demonstrated a significantly lower proportion of time spent sitting during class at 4 and 8 months. The proportion of time spent standing and the number of sit-to-stand transitions during class time were significantly greater in the intervention group at both 4 and 8 months, in comparison to the control group. The proportion of class time spent stepping was significantly greater in the intervention class at 8 months, but not at 4 months (Table 3). To account for differences in ethnic groups at baseline between classes (see Table 1), sensitivity analysis included South Asian ethnicity as a covariate within multi-level model analysis (see Supplementary Table S3). These models demonstrated reduced but comparable intervention effects during class time compared to the main analysis (Supplementary Table S3).

No significant differences were observed between groups in terms of the proportion of time spent sitting, standing, or stepping at 4 and 8 months after school, with the exception of a reduction in the proportion of time spent stepping after school in the intervention group, relative to controls, at 8 months (Table 3).

The intervention group demonstrated a significant reduction in the proportion of wear time spent sitting at 4 months and 8 months compared to the control group during a full weekday. Significant increases in the intervention group compared to the control group were observed in the proportion of wear time spent standing (4 and 8 months), and in sit-to-stand transitions per hour of wear time (4 and 8 months) during a full weekday. There were no differences observed in stepping time during a full weekday between groups at both follow-ups (Table 3). A more comprehensive set of sitting and physical activity outcomes within multi-level models are presented in Supplementary Table S2.

#### 3.2.2. Behavior-Related Mental Health, Musculoskeletal Discomfort, and Cognitive Function

Table 4 shows the estimated effect sizes of the intervention on behavior-related mental health, musculoskeletal discomfort, and cognitive function in the intervention group compared to the control group at both 4 and 8 months. There was a significant deterioration in behavior-related mental health measures in the intervention group relative to the control group at both 4 and 8 months. No intervention effects were observed in any musculoskeletal discomfort score variable at 4 and 8 months. The intervention group recorded a significantly slower reaction time in the Stroop test at 4 months compared to the control group, but no other differences were observed between groups (Table 4).

### 3.3. Qualitative Data

#### 3.3.1. Interviews and Focus Groups

A summary of participant’s responses to the questions that were asked regarding the standing desk intervention are provided below.

*“Do you think the standing desks are needed within your classroom and why?”*

The teacher stated that instead of an intervention to reduce sitting time, he would have preferred a physical education-based intervention to replace some English and Mathematics classes (the teacher was a physical education (PE) teacher in a previous occupation). The pupils stated that the desks are needed because “*they make you stand more which is good for your health and learning*”. They also stated that it “*feels more comfortable when you stand*”.

*“How well do you think you are able to teach/learn with the new standing desks?”*

The teacher stated that he had to adapt his teaching methods to cater for the new desks; he would normally have a different seating plan for each subject. However, due to the stools taking up so much space, this was not possible. He therefore had children sat in the same place all day and tailored his teaching and materials to each group of children. He also could not walk around the class as before; children had to come to him. Also, when children stood, on occasions other pupils bumped into them when moving around due to stools in the walkways.

The pupil’s comments included “*it is good to have the option to sit or stand*” because “*sitting can become uncomfortable*”. More than one child stated that standing can be more comfortable (than sitting) and can help with concentration. One negative point was that the new desks can be distracting. The reasons for this included “*because the new desks move around*” and that “*pupil’s move around the class more and can bump into you*” and “*nudge you while you work*”. Several pupils stated that “*it is better to stay in one place with your work instead of moving around like before*” whereas another student stated “*standing desks encourage standing and more moving around so less work gets done.*”

*“How have the desks affected the class atmosphere?”*

The teacher stated that overall behavior had improved but he felt this was due to the children remaining in one place from lesson to lesson and not because of an increase in standing. One pupil agreed that class behavior had improved because the children stay in the same place. However, one child stated that the class is “*noiser because they can talk about the desks*.”

#### 3.3.2. Classroom Observations

##### Observation One: Day 1 of Intervention

The researcher stated that during the 30 min observation, 3–6 children were standing and 3–4 children were “*perching*” (the remainder were sitting). When in the standing position, “*quite a few*” children were leaning on the desks instead of standing upright with a correct posture. Some children were also leaning excessively on one leg, causing increased spine curvature. When sitting down, the children tended to sit on the edge of the stool, so angles at the hip, knee, and ankle were greater than 90 degrees. Several children could not reach the floor with their feet. The researcher stated that after having brief conversations with teaching staff and pupils, in general the standing desks were liked but the teacher stated that the fit of the desks within the classroom was “*a little tight*.”

##### Observation Two: Week 16 (4 Months) of Intervention

During the second observation within the intervention class, the researcher described the following:

*“The children walk in from the morning assembly and the teacher immediately tells all children to raise their standing desks and begin working on the maths activities on the white board. There are a number of moans and groans from the children. It is clear this is a common practice. For the duration of ‘standing time’ a lot of children are leaning on the desks and are not actually standing up. A lot of the children kept sitting down on their stools. I counted the teacher telling individuals (not the same children) 10 times to stand back up during the 20-min period. Once the 20 min is complete and the teacher declares everyone can either sit or stand, 22 out of 27 children immediately chose to sit back down. After another five minutes two more children sit down, leading to three children standing. After a further five minutes two more children sit back down, leading to one child choosing to stand up to work. No other children chose to stand up during the remainder of the 30-min observation.”*

## 4. Discussion

This study aimed to explore the impact of allocating standing desks to every child within a Year 5 primary school classroom over an 8-month period on classroom sitting, physical activity, behavior-related mental health, musculoskeletal comfort, and markers of cognitive function. Overall, the standing desks influenced positive changes in sitting, standing, and sit-to-stand transitions during class time in the intervention group. The standing desks did not impact negatively on musculoskeletal discomfort; however, qualitative data revealed challenges to teaching practicalities, and there was a gradual decline in behavior-related mental health in the intervention group. The setting of this study is key; each school was located within a deprived neighborhood with high concentrations of South Asian children that are of higher risk of adverse health outcomes [37,44]. This setting is a priority for health interventions and therefore health enhancing intervention studies are essential. Larger trials, implemented within similar high-priority settings, and using more in-depth qualitative and quantitative measures are needed to better establish whether standing desks, using a full desk allocation system, are feasible or effective in UK primary schools.

The present study is the first in the UK to evaluate standing desks within the primary school classroom over the longer-term (>6 months). The intervention group demonstrated substantial reductions in class time sitting at both follow-ups (4 months: −96 min/−32% of wear time, 8 months: −60 min/−20% of wear time compared to baseline) in comparison to previous school-based full desk allocation studies where reductions of 44–65 min/day after 9 weeks [18,19] and 23 min/day after 8 months [18,22] have been observed. A reduction of 23 min/day after 8 months in the only previous longer-term study [18,22] is less than the change observed in the present study after 8 months (−60 min/day). This is despite sharing the same model of sit–stand desk, full allocation system, measurement of sitting time, comparable behavior change strategies (i.e., daily standing classes) and the children being similarly sedentary at baseline (68% and 70% during class time). The small samples and differences between countries and school environments are likely explanations for different sitting behaviors observed between studies. For example, the UK and Australia experience contrasting climates throughout a school year which may influence sitting, standing and stepping behaviors [18]. Furthermore, the study samples included different ethnic compositions and were set within neighborhoods of different socio-economic status (UK more deprived, Australia middle-upper class [18,22]; these are factors that can influence different sitting time and physical activity behaviors in children [37,38,60].

Interestingly, studies using partial desk allocation systems (several standing desks replace a few traditional desks and children are rotated) have observed comparable reductions in sitting to earlier full desk allocation studies despite less intervention exposure (i.e., 1 h per day); 52 min/day during class time after 9 weeks [18] and 26 min/day during school time after 3 months [61]. Existing evidence therefore suggests that changes in class time sitting between full desk allocation and partial desk allocation systems are relatively comparable. Consequently, it may be more cost-effective to prioritize partial desk allocation systems, and this should be evaluated in future fully powered full desk allocation and partial desk allocation comparison studies.

Reductions in class time sitting at the two follow-ups in the intervention group did not appear to result in a change in sitting behavior after school time. This suggests that compensatory changes after school did not occur and is in line with previous classroom-based standing desk studies [20,22]. activPAL data suggest an initial novelty effect after 4 months of exposure where the reduction in sitting time was greater at this measurement time-point (−32%) compared to at 8 months (−20%). Interestingly, the observed reluctance of the pupils to engage in the 20-min standing class after 4 months of exposure suggested that novelty effects had set in by this stage. Despite this, the extent of the reductions in sitting and standing/stepping outcomes at both follow-ups suggests that the intervention still enabled a positive change in sitting and activity behaviors after the initial excitement had dissipated (i.e., children were still engaging with the sit–stand desks). Interestingly, substantial changes were still apparent when deducting the enforced 20-min daily standing classes (4 months: −76 min/day, 8 months: −40 min/day compared to baseline). This suggests that pupils preferred to interrupt and replace sitting with standing when given the choice, rather than when standing was imposed upon them. The continuous exposure to the standing desk afforded by the full desk allocation system enabled children to choose when to sit or stand, likely increasing their autonomy. A key benefit of the full desk allocation system is the reduced demand on teaching staff to implement the intervention, in that teachers are not required to rotate children in their class to ensure equal exposure to a smaller number of standing desks

Interview and focus group data revealed issues with teaching methods. The predominant challenge to the practicalities of the full desk allocation system was restrictions in classroom space. When children stood at the standing desks, stools blocked the walkways, limiting space and forcing the teacher to adapt his teaching methods. The origin of this problem was the standing desk design, which meant that the stools could not be tucked underneath when not in use. Teacher and pupil interview data suggested that classroom behavior generally improved due to pupils remaining at the same desk throughout the school day. Nevertheless, while pupil movement around the classroom was minimized, the teacher and some pupils during focus groups suggested there were more “bumps” and “nudges” causing distractions and conflicts, perhaps explaining the contradictory decline in behavior-related mental health scores reported in the intervention group (this group moved from “close to average risk” to “slightly raised risk” of behavioral disorders after 8 months). If standing desk manufacturers are willing to take these findings on board and design standing desk models that are more space saving (e.g., stools stored underneath the desk), this may avoid such issues in the future.

Overall, few studies have explored the influence of standing desks on classroom behavior [20,21,23,31,62], and of which there is little evidence of a negative impact. There are previous examples, albeit scarce, of space issues in the classroom and conflict between children within standing desk classrooms. Within the “dynamic classroom” of the Aminian et al. [19] study, one child reported overcrowded shared workstations and conflict with demand for Swiss balls (for seated rests) as there was an insufficient number. Clemes et al. [18] also observed issues with insufficient classroom space from a partial desk standing intervention in a UK sample, but detrimental effects on behavior were not mentioned [18]. Importantly, there were no differences between groups in cognitive function outcomes in the present study (other than a slightly slower reaction time in the Stroop test in the intervention group). This suggests that standing desks were not detrimental to cognitive development over the longer-term, which is consistent with a previous full allocation standing desk study [21]. Recent evidence has shown limited links between sitting time, physical activity and cognitive function [4], suggesting the lack of changes seen herein were to be expected. Given the current evidence, it may therefore be inappropriate to evaluate cognitive function outcomes in future classroom-based standing desk intervention studies. However, in general, impacts on development and learning still need to be accounted for as these outcomes will be of priority to schools and parents.

Class observation data showed pupils adopted incorrect postures when standing at the desks (i.e., excessive leaning onto the desk or leaning to one side). There is evidence that a poor standing posture can lead to acute lower back pain, albeit in adults [63]. No differences were observed between groups in musculoskeletal discomfort scores, even after 8 months. Despite this, it is possible these poor postures may lead to musculoskeletal issues over the longer-term. In adolescents, there is evidence of self-reported discomfort in the back or legs after 7-weeks of using sit–stand desks in the classroom [64] whereas a more recent study reported no changes in discomfort after 17 weeks of sit–stand desk exposure [17]. It was concluded that instructions provided to pupils and teacher on correct posture, only provided in the latter study, may explain these different findings [17]. However, neither study used classroom observations to capture adopted postures. In the present study, correct desk usage was demonstrated to pupils by researchers and posters highlighting correct postures were placed around the classroom; however, poor postures were still observed. Clearly more efforts were needed in the present study. More longer-term studies using frequent classroom observations are needed in children and adolescents to better understand the relationship between posture when using standing desks and musculoskeletal health during different stages of physical development.

Strengths of this study include the setting which consisted of priority demographics for reducing health inequalities (children from ethnic minorities and of low socio-economic position). Furthermore, the intervention spanned almost an entire academic year with two follow up phases and the control group was located in a separate school to avoid contamination [18,22]. The study also included a more holistic evaluation approach using mixed-method approaches and was novel in implementing a full desk allocation system in the UK. Study limitations include the small sample of schools and classes, limiting the external validity of the findings, the poor activPAL compliance, particularly at the follow up phases, and the absence of validity data supporting the use of the musculoskeletal discomfort questionnaire. Interviews and focus groups should have been audio recorded to enable more robust and in-depth analysis. Therefore, these qualitative findings should be interpreted with caution. Socio-economic position could not be controlled for as a confounder in the intervention impact analysis as individual level data were not collected, although the neighborhood-level socio-economic position of the two schools was comparable. Just two classroom observations will not have provided full insight into standing desk engagement; however, the research team were cautious of over burdening the participants and teaching staff with study measurements. Finally, there were substantially more South Asian children in the control group compared to the intervention group; however, supplementary analysis demonstrated consistent findings with the main analysis.

## 5. Conclusions

The findings suggest providing sit–stand desks to every child within a UK primary school classroom can reduce class time sitting throughout most of an academic year. Furthermore, positive changes were observed in standing and sit-to-stand transitions during class time at both follow-ups. This study was located within a deprived setting with a high proportion of ethnic minorities, making the findings more important in relation to reducing health inequalities. The sit–stand desks did not impact negatively on musculoskeletal discomfort, or cognitive function, and were generally well tolerated by pupils and staff. However, there were challenges to teaching practicalities and a gradual decline in behavior-related mental health was observed. Larger trials, implemented within similar high-priority settings, and using more in-depth qualitative and quantitative measures are needed to better establish whether standing desks using a full desk allocation system are feasible, or effective in UK primary schools. Future full desk allocation studies will, however, depend on the balance between the desired level of standing desk provision (full vs. partial allocation), class size, and available budgets.

## Figures and Tables

**Table 1:** Comparison of baseline characteristics between the control and intervention groups. Data presented as mean (SD) unless stated otherwise.

Descriptive Characteristics	Control	Intervention	*p*-Value INT vs. Control
*N*	27	22	
Age, years	9.7 (0.4)	9.8 (0.3)	0.66
Boys, %	46.2	50.0	0.79
Ethnicity			
South Asian, *N* (%)	24 (88.5)	10 (45.5)	**<0.01**
White British, *N* (%)	2 (7.7)	11 (50)	**<0.01**
Other, *N* (%)	1 (3.8)	1 (4.5)	0.90
BMI z-score (kg/m^2^)	0.40 (1.35)	0.34 (1.42)	0.90
BMI categories (%)			
% Underweight	3.9	9.1	0.45
% Normal	61.5	63.6	0.88
% Overweight	11.5	13.6	0.83
% Obese	23.1	18.2	0.67
**activPAL data**			
Valid weekdays, *N* †	6.0 (2.0)	5.0 (0.3)	0.23
Class time wear time, mins/day †	309.9 (21.3)	305.0 (5.5)	**<0.01**
Class time sitting, % of wear time	73.9 (1.8)	70.4 (2.9)	0.28
Class time standing, % of wear time	17.2 (7.6)	21.5 (10.9)	0.11
Class time stepping, % of wear time †	9.0 (2.8)	8.8 (2.8)	0.57
Class time sit-to-stand transitions, p/h of wear time	7.1 (2.5)	8.4 (3.0)	0.09
After school wear time, mins/day	394.6 (42.6)	424.2 (26.4)	0.07
After school sitting, % of wear time	69.7 (11.7)	70.6 (7.1)	0.74
Full day wear time, mins/day †	892.1 (59.2)	942.0 (40.9)	**<0.01**
Full day sitting, % of wear time	72.9 (3.8)	68.3 (1.9)	0.32
**Behavior-related mental health**			
Total score, max score of 40	7.6 (5.1)	9.6 (5.9)	0.22
**Musculoskeletal discomfort**			
Whole body, mean of all scales *	1.9 (1.8, 2.2)	1.8 (1.5, 2.1)	0.40
Upper limb, sum score †	4.0 (2.0)	3.0 (2.0)	0.07
Neck and back, sum score †	4.0 (3.0)	3.0 (2.0)	0.43
Lower limb, sum score	6.5 (0.5)	6.3 (0.5)	0.76
**Cognitive function**			
Stroop test, reaction time (ms) † Control *N* = 20, intervention *N* = 20	497.0 (239.0)	441.5 (235.0)	0.82
Corsi Block Tapping test, score out of 12 Control *N* = 25, intervention *N* = 22	4.2 (1.4)	4.4 (1.3)	0.52

*p*-values are obtained using two-sample *t*-tests or Pearson chi-square tests as appropriate. † Data represent the median and interquartile ranges due to skewed distributions that were not corrected after transformations. The Wilcoxon signed-rank test was used to compare values. * Mean value and confidence intervals taken from log transformed data which were then back transformed. Data compared using independent *t*-tests. INT, intervention, BMI, body mass index.

**Table 2:** Data compliance at each measurement time-point, based on the proportion of participants who wore activPAL monitors at baseline.

	Control ^a^						Intervention ^b^					
	Baseline		4 Months		8 Months		Baseline		4 Months		8 Months	
	%	*N*	%	*N*	%	*N*	%	*N*	%	*N*	%	*N*
**activPAL**	96	27	75	21	79	22	81	22	63	17	56	15
**Behavior-related mental health**	96	27	81	26	89	25	81	22	74	20	81	22
**Musculoskeletal discomfort**	96	27	81	25	93	26	81	22	81	22	78	21
**Cognitive function**	71	20	74	18	68	19	74	20	63	17	63	17

^a^ 28 participants wore activPAL monitors at baseline; ^b^ 27 participants wore activPAL monitors at baseline.

**Table 3:** Estimated effect sizes of the intervention in activPAL-determined outcomes during class time, after school and full weekdays at baseline, 4 months, and 8 months from multi-level models.

	Time-Point								
	Baseline			4 Months			8 Months		
Outcome	β	95% CI	*p*	β	95% CI	*p*	β	95% CI	*p*
**Class time**									
Wear time	−9.80	(−17.01,−2.58)	**0.008**	−21.99	(−30.18,−13.81)	**0.001**	−11.39	(−19.79,−2.99)	**0.008**
Sitting time, % of wear time	−3.57	(−9.83,2.70)	0.265	−25.34	(−32.25,−18.43)	**0.001**	−19.99	(−27.05,−12.94)	**0.001**
Standing time, % of wear time	4.36	(−0.96,9.68)	0.108	25.74	(19.91,31.58)	**0.001**	17.82	(11.88,23.76)	**0.001**
Stepping time, % of wear time	−0.81	(−2.62,1.01)	0.384	−0.26	(−2.28,1.75)	0.798	2.21	(0.15,4.27)	**0.035**
Sit-to-stand transitions, p/h wear time	1.37	(−0.05,2.80)	0.058	2.92	(1.33,4.51)	**0.001**	4.62	(2.99,6.24)	**0.001**
**After school**									
Sitting, % of wear time	0.97	(−4.74,6.68)	0.739	3.7	(−2.50,9.90)	0.242	1.29	(−5.17,7.75)	0.696
Standing, % WT	1.07	(−0.88,3.02)	0.283	−0.25	(−3.72,3.22)	0.887	3.55	(−0.37,7.48)	0.076
Stepping, % WT	0.61	(−1.28,2.49)	0.529	−2.30	(−5.58,0.98)	0.169	−3.73	(−0.03,−7.43)	**0.048**
**Full Day**									
Wear time, mins	59.9	(17.79,102.02)	**0.005**	7.49	(−38.94,53.93)	0.752	12.48	(−35.93,60.89)	0.613
Sitting time, % of wear time	−1.00	(−5.69,3.69)	0.675	−7.67	(−12.77,−2.57)	**0.003**	−5.52	(−10.84,−0.19)	**0.042**
Standing time, % of wear time	1.3	(−2.07,4.68)	0.45	5.78	(2.03,9.53)	**0.003**	8.78	(5.16,12.40)	**0.001**
Stepping time, % of wear time	−0.29	(−2.62,2.03)	0.805	−0.87	(−3.38,1.65)	0.498	−0.20	(−2.81,2.42)	0.883
Sit-to-stand transitions, p/hr wear time	0.6	(−0.36,1.56)	0.222	1.44	(0.36,2.52)	**0.009**	1.36	(0.29,2.43)	**0.013**

p/min, per minute; p/hr, per hour, WT, wear time.

**Table 4:** Estimated effect sizes of the intervention in behavior-related mental health, musculoskeletal discomfort, and cognitive function at baseline, 4 months, and 8 months from multi-level models. Control *n* = 27, Intervention *n* = 22.

	Time-Point								
	Baseline			4 Months			8 Months		
Outcome	β	95% CI	*p*	β	95% CI	*p*	B	95% CI	*p*
**Behavior-related mental health**									
Total score	2.06	(−0.65,4.78)	0.136	5.31	(2.55,8.08)	**0.001**	7.92	(5.18,10.66)	**0.001**
**Musculoskeletal discomfort**									
Whole body	−0.08	(−0.43,0.26)	0.632	−0.27	(−0.62,0.08)	0.132	−0.07	(−0.42,0.29)	0.710
Upper Limb, combined score	−0.41	(−1.11,0.30)	0.262	−0.38	(−1.10,0.35)	0.310	−0.08	(−0.81,0.64)	0.818
Neck and back, combined score	−0.23	(−1.14,0.68)	0.618	−0.79	(−1.72,0.15)	0.099	−0.09	(−1.02,0.84)	0.851
Lower Limb, combined score	−0.13	(−1.55,1.28)	0.852	−0.39	(−1.85,1.07)	0.603	0.08	(−1.36,1.53)	0.911
**Cognitive function**									
Corsi Block Tapping Control *N* = 20, INT *N* = 20)	0.21	(−0.52,0.94)	0.573	−0.33	(−1.08,0.43)	0.398	0.11	(−0.64,0.86)	0.769
Stroop, reaction time (Control *N* = 25, INT *N* = 22)	25.43	(−97.35,148.22)	0.685	133.67	(3.72,263.62)	**0.044**	37.37	(−92.58,167.32)	0.573

INT, intervention.

## Supplementary Materials

The following are available online at https://www.mdpi.com/1660-4601/17/19/7048/s1. Table S1: Sitting, standing and stepping outcomes in control and full desk allocation groups during class time and school breaks at baseline, 4 months and 8 months. Data presented as median minutes (interquartile range), and the median (interquartile range) proportion of wear time spent in each behaviour during different domains, Table S2: Estimated effect sizes of the intervention in activPAL-determined outcomes during class time, after school and full weekdays at baseline, 4 months and 8 months from multi-level models, Table S3: A comparison of sitting, standing and stepping outcomes during different times of a weekday between multi-level models with and without South Asian ethnicity as a covariate, File S1: Planned focus group and interview questions, File S2: Multi-level modelling methodology.
