# Supplementary material for: Impacts of a Standing Desk Intervention within an English Primary School Classroom: A Pilot Controlled Trial
Source: Int J Environ Res Public Health. 2020 Sep 26;17(19):7048. doi: 10.3390/ijerph17197048 (PMC7579086; doi:10.3390/ijerph17197048)
Supplement: Supplementary file 1 [file ijerph-17-07048-s001.zip › Supplementary file 1. Planned focus group and interview questions.docx]

**File S1: Planned focus group and interview questions**

Semi-structure interview and focus group questions were based on Durlak and DuPre’s (1) summary of factors that affect intervention implementation. Questions were generated using factors that were interpreted as most relevant to a classroom-based standing desk intervention. The item labels below correspond with items within Durlak and DuPre summary list (see page 3). Questions that were for pupils or teachers only are indicated at the end of each item.

**II**

A – Do you think the new desks are needed? Why?

B– How relevant are the desks to the school needs? (teacher only)

B – What do you think the potential benefits of the desks are?

C – How well do you think you are able to learn/teach with these desks?

**III**

A – How well do the desks fit with the school and pupils needs? (teacher only)

B – To what extent can the desks be used/adapted to fit with the school and pupils needs? (teacher only)

**IV**

A1 – How have the desks affected the class atmosphere?

A2 – How have the pupils adapted to the desks? How willing are the pupils to change their sitting and standing behaviour? (teacher only)

A3 – How have the desks changed your learning and class experience? (Pupils only)

A4 – Are you aware of the purpose of the desks? Do you see this as important or relevant for to? (pupils only)

B1 – Have other teaching staff been consulted on the implementation of teaching with these desks and how to increase standing time (i.e. sedentary behaviour reduction strategies). (teacher only)

B3 – Have correct postures been discussed with you (pupil)/teaching assistants (teacher)?

C1 – Are there class champions for correct postures?

**V**

A – Do you think you have had sufficient instruction and training with the standing desks to correctly use them in your class? (teacher only)

B – Is there sufficient support from research team members for you to implement the desks effectively? (teacher only)

Durlack and DuPre (2008): factors affecting the implementation process.

**
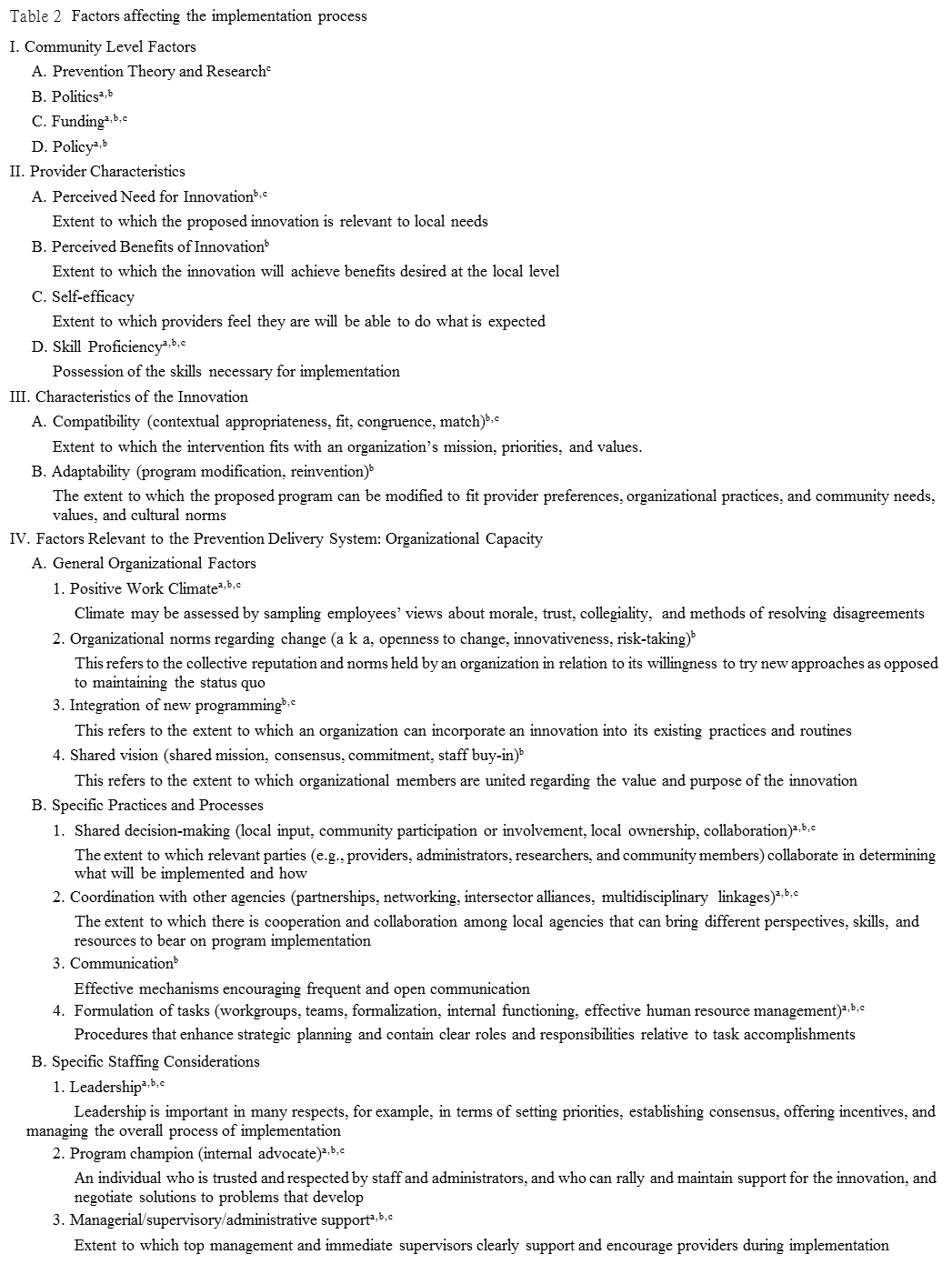
**

**REFERENCES**

1. Durlak JA, Dupre ÆEP. Implementation Matters : A Review of Research on the Influence of Implementation on Program Outcomes and the Factors Affecting Implementation. *American Journal of Community Psychology*. 2008;327–50.
