# Supplementary material for: Impacts of a Standing Desk Intervention within an English Primary School Classroom: A Pilot Controlled Trial
Source: Int J Environ Res Public Health. 2020 Sep 26;17(19):7048. doi: 10.3390/ijerph17197048 (PMC7579086; doi:10.3390/ijerph17197048)
Supplement: Supplementary file 1 [file ijerph-17-07048-s001.zip › Supplementary file 2. Multilevel modelling methodology.docx]

**File S2: Multi-level modelling methodology**

The following equation was applied in the modelling:

y^it^ = B^oi^ + B^1^time point ^2^ + B^2^time point ^3^ + B^3^ condition + B^4^Time point^2^ X condition + B^5^ Time^3^ X condition + E^it^

B0i = B0 + U0i

U^i^ ~ N(0,σ  )

E^it^ ~ N(0,σ  )

In this equation, y^it^ is the outcome in individual i at time point t, B^oi^ is an intercept comprising a sample-average fixed effect (B) and a level 2 (i.e., individual) random effect (U), B^1^ and B^2^ are binary dummy terms indicating the time point (0=no, 1=yes), B^3^ is a binary term indicating the intervention (0=control group, 1=intervention group) and B^4^ & B^5^ are the interaction between time point and intervention. E^ij^ is a level 1 (measurement occasion) random effect capturing residual error. The U^i^ and E^ij^ are assumed to be independent and normally distributed with zero means and variances (U^i^ ~ N(0,σ  ), E^it^ ~ N(0,σ  ))

Within each dependent variable of interest (e.g. proportion of wear time spent sitting during class time), visual data checks were performed in the frequency of observations at each time point in each condition to determine whether any missing data were systematic. If missing data were not interpreted as systematic, models were then built. Firstly, a variance component model (with no explanatory variables included) was built. The variance partitioning coefficient (VPC) statistic was used to determine how much variance is explained by each of the two levels (measurement occasion and individual). A second model was then built that included time points (4 months and 8 months), condition and time-point-by-condition interactions as explanatory variables (see full equation above). Covariates were not included in models due to CONSORT guidelines recommending the use of unadjusted models within trial studies (1). However, since randomisation was not carried out in this trial, where applicable, sensitivity analysis was conducted using variables that were different at baseline between groups as covariates (see supplementary Table S3).

Level 1 (measurement occasion) and level 2 (individual) residuals were obtained and histograms plotted to explore distributions (normal distribution of residuals is an assumption of the model). Level 1 residuals were then further explored in an attempt to reduce model error; scatter plots were inspected to determine high residual values that suggest poor fitting trajectories of individuals. Participants with high residuals were identified and their original values (i.e. proportion of wear time spent sitting at baseline, 4 months and 8 months) were inspected to observe how these values compare. High residual values were then either deleted or remained within the analysis, depending on the nature of the outcome variable and previous research. For example, sitting and physical activity behaviour can be somewhat variable from day-to-day (2) and between different seasons of the year in children (3) and therefore considerable tolerance was allowed for high residuals in these outcomes. Other information within the data, if available, was also used to help inform the decision. For example, a low proportion of wear time spent sitting at baseline could be cross-checked with accumulated sitting minutes and standing and stepping data at baseline to determine whether the values combine coherently. If any observations were removed from the analysis, models were then re-fitted, and a comparison of model accuracy was made. These checks include the Wald statistic, the likelihood ratio test, level 1 variance, and the Bayesian Information Criterion (BIC). The Wald statistic should be significant (*P <*0.05) and the latter three indicators should produce lower values if the new model is a better fit. The level of significance for determining an intervention effect was set at (*P <*0.05).

**References**

1. Moher D, Hopewell S, Schulz KF, Montori V, Gøtzsche PC, Devereaux PJ, et al. & reporting CONSORT 2010 Explanation and Elaboration : updated guidelines for reporting parallel group randomised trials. BMJ. 2010;340:869.

2. Ridgers ND, Timperio A, Cerin E, Salmon J. Within- and between-day associations between children ’ s sitting and physical activity time. BMC Public Health [Internet]. BMC Public Health; 2015;15:1–7. Available from: http://dx.doi.org/10.1186/s12889-015-2291-3

3. King AC, Parkinson KN, Adamson AJ, Murray L, Besson H, Reilly JJ, et al. Correlates of objectively measured physical activity and sedentary behaviour in English children. Eur J Public Health. 2010;21(4):424–31.
