# Supplementary material for: Impacts of a Standing Desk Intervention within an English Primary School Classroom: A Pilot Controlled Trial
Source: Int J Environ Res Public Health. 2020 Sep 26;17(19):7048. doi: 10.3390/ijerph17197048 (PMC7579086; doi:10.3390/ijerph17197048)
Supplement: Supplementary file 1 [file ijerph-17-07048-s001.zip › Supplementary Tables S1, S2, S3.docx]

**Table S1**. Sitting, standing and stepping outcomes in control and full desk allocation groups during class time and school breaks at baseline, 4 months and 8 months. Data presented as median minutes (interquartile range), and the median (interquartile range) proportion of wear time spent in each behaviour during different domains.

|  | **Control (**n=**27)** | | | | | | | | | **Intervention (**n=**22)** | | | | | | | | |  |
| --- | --- | --- | --- | --- | --- | --- | --- | --- | --- | --- | --- | --- | --- | --- | --- | --- | --- | --- | --- |
|  | **Baseline** | | **4 Months** | | | **▲ Vs B** | **8 Months** | | **▲ Vs B** | **Baseline** | | **4 Months** | | **▲ Vs B** | **8 Months** | | **▲ Vs B** | | |
| **Class time** |  |  | |  |  |  |  |  |  |  |  |  |  |  |  |  | |  | |
| WT, mins | 309.9 | (21.3) | | 330.0 | (0.0) | 20.1 | 308.2 | (20.7) | -1.7 | 305.0 | (5.5) | 305.0 | (0.0) | 0.0 | 305.0 | (2.6) | | 0.0 | |
| Sitting, mins | 232.2 | (40.9) | | 238.7 | (54.0) | 6.5 | 216.8 | (41.9) | -15.4 | 218.5 | (58.7) | 122.6 | (68.0) | -95.9 | 158.5 | (73.2) | | -60.0 | |
| Sitting, % WT | 73.7 | (10.2) | | 72.6 | (13.4) | -1.1 | 72.1 | (6.6) | -1.6 | 72.2 | (19.3) | 40.5 | (23.3) | -31.7 | 52.4 | (21.9) | | -19.8 | |
| Standing, % WT | 16.6 | (8.4) | | 16.3 | (8.5) | -0.3 | 17.6 | (9.0) | 1.0 | 19.2 | (16.6) | 43.5 | (21.1) | 24.3 | 35.6 | (18.1) | | 16.4 | |
| Stepping, % WT | 9.0 | (2.8) | | 11.1 | (4.6) | 2.1 | 11.0 | (2.7) | 2.0 | 8.8 | (2.8) | 11.2 | (5.1) | 2.4 | 12.0 | (4.0) | | 3.2 | |
| Steps p/min WT | 6.9 | (2.0) | | 8.6 | (3.6) | 1.7 | 8.5 | (2.5) | 1.6 | 6.3 | (1.4) | 7.0 | (2.9) | 0.7 | 8.8 | (3.3) | | 2.5 | |
| SIT2STD Trans p/h WT | 7.1 | (3.0) | | 7.8 | (2.0) | 0.7 | 5.6 | (2.2) | -1.5 | 8.2 | (3.6) | 11.2 | (5.4) | 3.0 | 10.7 | (2.3) | | 2.5 | |
| *5-10-minute bouts* |  |  | |  |  |  |  |  |  |  |  |  |  |  |  |  | |  | |
| Sitting, % WT | 11.5 | (6.9) | | 10.1 | (5.5) | -1.4 | 8.9 | (4.0) | -2.6 | 13.6 | (5.8) | 5.2 | (5.2) | -8.4 | 6.4 | (5.6) | | -7.2 | |
| *10+ minute bouts* |  |  | |  |  |  |  |  |  |  |  |  |  |  |  |  | |  | |
| Sitting, % WT | 40.9 | (13.3) | | 43.5 | (22.3) | 2.6 | 49.4 | (16.0) | 8.5 | 35.5 | (18.5) | 19.3 | (7.5) | -16.2 | 18.7 | (12.7) | | -16.8 | |
| **After School** |  |  | |  |  |  |  |  |  |  |  |  |  |  |  |  | |  | |
| WT, mins | 395.8 | (52.0) | | 403.2 | (99.6) | 7.4 | 435.5 | (52.6) | 39.7 | 422.4 | (34.3) | 408.7 | (66.8) | -13.7 | 424.4 | (93.8) | | 2.0 | |
| Sitting, mins | 276.0 | (55.6) | | 237.5 | (86.4) | -38.5 | 254.5 | (83.5) | -21.5 | 293.9 | (56.0) | 270.9 | (76.2) | -23.0 | 269.4 | (90.2) | | -24.5 | |
| Sitting, % WT | 69.4 | (11.8) | | 62.1 | (14.1) | -7.3 | 59.1 | (15.3) | -10.3 | 69.6 | (12.2) | 69.2 | (14.2) | -0.4 | 64.4 | (9.0) | | -5.2 | |
| **Full weekday** |  |  | |  |  |  |  |  |  |  |  |  |  |  |  |  | |  | |
| WT, mins | 892.1 | (59.1) | | 948.8 | (110.3) | 56.7 | 924.6 | (68.5) | 32.5 | 942.0 | (40.9) | 933.8 | (117.4) | -8.2 | 968.4 | (114.6) | | 26.4 | |
| Sitting, mins | 617.9 | (105.2) | | 566.3 | (123.0) | -51.6 | 599.4 | (322.8) | -18.5 | 637.7 | (80.9) | 530.3 | (140.1) | -107.4 | 570.5 | (94.4) | | -67.2 | |
| Sitting, % WT | 69.5 | (10.5) | | 65.4 | (12.3) | -4.1 | 63.5 | (9.7) | -6.0 | 68.1 | (10.6) | 57.4 | (7.8) | -10.7 | 59.1 | (10.3) | | -9.0 | |

**▲** Change; B, baseline; SIT2STD Trans, sit-to-stand transitions; p/h, per hour; WT, wear time

**Table S2**. Estimated effect sizes of the intervention in activPAL-determined outcomes during class time, after school and full weekdays at baseline, 4 months and 8 months from multi-level models.

|  | **Time point** | | | | | | | | | | | | |
| --- | --- | --- | --- | --- | --- | --- | --- | --- | --- | --- | --- | --- | --- |
|  | **Baseline** | | | | **4 Months** | | | | | **8 Months** | | | |
| **Outcome** | **β** | **95% CI** | | ***P*** | **β** | **95% CI** | | ***P*** | **β** | | **95% CI** | | ***P*** |
| **Class time** |  |  |  |  |  |  |  |  |  | |  |  |  |
| Wear time | -9.80 | (-17.01, | -2.58) | **0.008** | -21.99 | (-30.18, | -13.81) | **0.001** | -11.39 | | (-19.79, | -2.99) | **0.008** |
| Sitting time, % of wear time | -3.57 | (-9.83, | 2.70) | 0.265 | -25.34 | (-32.25, | -18.43) | **0.001** | -19.99 | | (-27.05, | -12.94) | **0.001** |
| Standing time, % of wear time | 4.36 | (-0.96, | 9.68) | 0.108 | 25.74 | (19.91, | 31.58) | **0.001** | 17.82 | | (11.88, | 23.76) | **0.001** |
| Stepping time, % of wear time | -0.81 | (-2.62, | 1.01) | 0.384 | -0.26 | (-2.28, | 1.75) | 0.798 | 2.21 | | (0.15, | 4.27) | **0.035** |
| Sitting time in 10+ min bouts, % of wear time | -3.31 | (-9.40, | 2.79) | 0.288 | -17.35 | (-24.04, | -10.66) | **0.001** | -28.96 | | (-35.81, | -22.10) | **0.001** |
| Sitting time in 5-10min bouts, % of wear time | 2.39 | (0.02, | 4.75) | **0.048** | -5.29 | (-7.87, | -2.70) | **0.001** | -0.61 | | (-3.25, | 2.04) | 0.653 |
| Steps, p/min of wear time | -1.16 | (-2.41, | 0.09) | 0.068 | -1.64 | (-3.04, | -0.24) | **0.021** | 0.86 | | (-0.57, | 2.29) | 0.238 |
| Sit-to-stand transitions, p/h wear time | 1.37 | (-0.05, | 2.80) | 0.058 | 2.92 | (1.33, | 4.51) | **0.001** | 4.62 | | (2.99, | 6.24) | **0.001** |
| **After school** |  |  |  |  |  |  |  |  |  | |  |  |  |
| Sitting, % of wear time | 0.97 | (-4.74, | 6.68) | 0.739 | 3.70 | (-2.50, | 9.90) | 0.242 | 1.29 | | (-5.17, | 7.75) | 0.696 |
| Standing, % of wear time | 1.07 | (-0.88 | 3.02) | 0.283 | -0.25 | (-3.72 | 3.22) | 0.887 | 3.55 | | (-0.37 | 7.48) | 0.076 |
| Stepping, % of wear time | 0.61 | (-1.28 | 2.49) | 0.529 | -2.30 | (-5.58 | 0.98) | 0.169 | -3.73 | | (-0.03 | -7.43) | **0.048** |
| **Full Day** |  |  |  |  |  |  |  |  |  | |  |  |  |
| Wear time, mins | 59.90 | (17.79, | 102.02) | **0.005** | 7.49 | (-38.94, | 53.93) | 0.752 | 12.48 | | (-35.93, | 60.89) | 0.613 |
| Sitting time, % of wear time | -1.00 | (-5.69, | 3.69) | 0.675 | -7.67 | (-12.77, | -2.57) | **0.003** | -5.52 | | (-10.84, | -0.19) | **0.042** |
| Standing time, % of wear time | 1.30 | (-2.07, | 4.68) | 0.450 | 5.78 | (2.03, | 9.53) | **0.003** | 8.78 | | (5.16, | 12.40) | **0.001** |
| Stepping time, % of wear time | -0.29 | (-2.62, | 2.03) | 0.805 | -0.87 | (-3.38, | 1.65) | 0.498 | -0.20 | | (-2.81, | 2.42) | 0.883 |
| Sitting time in 10+min bouts, % of wear time | -0.53 | (-4.85, | 3.79) | 0.811 | -3.96 | (-8.63, | 0.72) | 0.097 | -7.40 | | (-12.28, | -2.52) | **0.003** |
| Sitting time in 5-10min bouts, % of wear time | 0.93 | (-0.60, | 2.45) | 0.234 | -1.78 | (-3.42, | -0.15) | **0.032** | -0.33 | | (-2.03, | 1.36) | 0.699 |
| Steps, p/min of wear time | -0.45 | (-2.39, | 1.49) | 0.647 | -0.90 | (-3.08, | 1.28) | 0.419 | -0.35 | | (-2.54, | 1.84) | 0.753 |
| Sit-to-stand transitions, p/hr wear time | 0.60 | (-0.36, | 1.56) | 0.222 | 1.44 | (0.36, | 2.52) | **0.009** | 1.36 | | (0.29, | 2.43) | **0.013** |

p/min, per minute; p/hr, per hour

**Table S3.** A comparison of sitting, standing and stepping outcomes during different times of a weekday between multi-level models with and without South Asian ethnicity as a covariate.

|  |  |  |  |  | **Time point** |  |  |  |  |
| --- | --- | --- | --- | --- | --- | --- | --- | --- | --- |
|  |  | **Baseline** |  |  | **4 Months** |  |  | **8 Months** |  |
| **Outcome** | **β** | **95% CI** | ***P*** | **β** | **95% CI** | ***P*** | **β** | **95% CI** | ***P*** |
| **Class time**  Sitting time, % of wear time | -3.57 | (-9.83, 2.70) | 0.265 | -25.34 | (-32.25, -18.43) | 0.001 | -19.99 | (-27.05, -12.94) | 0.001 |
| South Asian covariate | -1.71 | (-8.29 4.87) | 0.611 | -21.72 | (-28.90 -14.54) | 0.001 | -16.33 | (-23.64 -9.01) | 0.001 |
| Standing time, % of wear time | 4.36 | (-0.96, 9.68) | 0.108 | 25.74 | (19.91, 31.58) | 0.001 | 17.82 | (11.88, 23.76) | 0.001 |

| South Asian covariate | 2.90 | (-2.73 | 8.53) | 0.312 | 21.35 | (15.57 | 27.14) | 0.001 | 13.39 | (7.49 | 19.29) | 0.001 |
| --- | --- | --- | --- | --- | --- | --- | --- | --- | --- | --- | --- | --- |
| Stepping time, % of wear time | -0.81 | (-2.62, | 1.01) | 0.384 | -0.26 | (-2.28, | 1.75) | 0.798 | 2.21 | (0.15, | 4.27) | 0.035 |
| South Asian covariate | -1.20 | (-3.12 | 0.71) | 0.218 | 0.54 | (-1.65 | 2.74) | 0.629 | 2.99 | (0.75 | 5.23) | 0.009 |
| Sitting time in 10+ min bouts, % of wear time | -3.31 | (-9.40, | 2.79) | 0.288 | -17.35 | (-24.04, | -10.66) | 0.001 | -28.96 | (-35.81, | -22.10) | 0.001 |
| South Asian covariate | -1.87 | (-8.24 | 4.50) | 0.565 | -14.02 | (-22.00 | -6.03) | 0.001 | -25.55 | (-33.65 | -17.46) | 0.001 |
| Sitting time in 5-10min bouts, % of wear time | 2.39 | (0.02, | 4.75) | 0.048 | -5.29 | (-7.87, | -2.70) | 0.001 | -0.61 | (-3.25, | 2.04) | 0.653 |
| South Asian covariate | 2.47 | (-0.03 | 4.97) | 0.053 | -7.67 | (-10.69 | -4.66) | 0.001 | -2.99 | (-6.05 | 0.07) | 0.056 |
| Steps, p/min of wear time | -1.16 | (-2.41, | 0.09) | 0.068 | -1.64 | (-3.04, | -0.24) | 0.021 | 0.86 | (-0.57, | 2.29) | 0.238 |
| South Asian covariate | -1.36 | (-2.68 | -0.04) | 0.044 | -0.48 | (-2.07 | 1.11) | 0.552 | 2.01 | (0.39 | 3.63) | 0.015 |
| Sit-to-stand transitions, p/hr wear time | 1.37 | (-0.05, | 2.80) | 0.058 | 2.92 | (1.33, | 4.51) | 0.001 | 4.62 | (2.99, | 6.24) | 0.001 |
| South Asian covariate | 1.17 | (-0.34 | 2.68) | 0.127 | 1.55 | (-0.23 | 3.33) | 0.088 | 3.23 | (1.42 | 5.04) | 0.001 |
| **After school**  Sitting, % of wear time | 0.97 | (-4.74, | 6.68) | 0.739 | 3.70 | (-2.50, | 9.90) | 0.242 | 1.29 | (-5.17, | 7.75) | 0.696 |
| South Asian covariate | 0.27 | (-5.82 | 6.35) | 0.932 | 2.76 | (-3.87 | 9.38) | 0.415 | 0.28 | (-6.59 | 7.15) | 0.937 |
| **Full Day** |  |  |  |  |  |  |  |  |  |  |  |  |
| Sitting time, % of wear time | -1.00 | (-5.69, | 3.69) | 0.675 | -7.67 | (-12.77, | -2.57) | 0.003 | -5.52 | (-10.84, | -0.19) | 0.042 |
| South Asian covariate | -0.39 | (-5.37 | 4.60) | 0.880 | -6.68 | (-12.23 | -1.13) | 0.018 | -4.47 | (-10.23 | 1.29) | 0.128 |
| Standing time, % of wear time | 1.30 | (-2.07, | 4.68) | 0.450 | 5.78 | (2.03, | 9.53) | 0.003 | 8.78 | (5.16, | 12.40) | 0.001 |
| South Asian covariate | 0.60 | (-3.01 | 4.20) | 0.746 | 7.49 | (3.99 | 10.99) | 0.001 | 4.45 | (0.81 | 8.08) | 0.017 |
| Stepping time, % of wear time | -0.29 | (-2.62, | 2.03) | 0.805 | -0.87 | (-3.38, | 1.65) | 0.498 | -0.20 | (-2.81, | 2.42) | 0.883 |
| South Asian covariate | -0.21 | (-2.71 | 2.28) | 0.866 | -0.58 | (-3.17 | 2.01) | 0.662 | 0.10 | (-2.59 | 2.79) | 0.070 |
| Sitting time in 10+ min bouts, % of wear time | -0.53 | (-4.85, | 3.79) | 0.811 | -3.96 | (-8.63, | 0.72) | 0.097 | -7.40 | (-12.28, | -2.52) | 0.003 |
| South Asian covariate | -1.87 | (-8.24 | 4.50) | 0.565 | -14.02 | (-22.00 | -6.03) | 0.001 | -25.55 | (-33.65 | -17.46) | 0.001 |
| Sitting time in 5-10min bouts, % of wear time | 0.93 | (-0.60, | 2.45) | 0.234 | -1.78 | (-3.42, | -0.15) | 0.032 | -0.33 | (-2.03, | 1.36) | 0.699 |
| South Asian covariate | 2.47 | (-0.03 | 4.97) | 0.053 | -7.67 | (-10.69 | -4.66) | 0.000 | -2.99 | (-6.05 | 0.07) | 0.056 |
| Steps, p/min of wear time | -0.45 | (-2.39, | 1.49) | 0.647 | -0.90 | (-3.08, | 1.28) | 0.419 | -0.35 | (-2.54, | 1.84) | 0.753 |
| South Asian covariate | -0.48 | (-2.56 | 1.60) | 0.650 | -0.45 | (-2.75 | 1.85) | 0.703 | 0.10 | -2.21 | 2.41) | 0.933 |
| Sit-to-stand transitions, p/hr wear time | 0.60 | (-0.36, | 1.56) | 0.222 | 1.44 | (0.36, | 2.52) | 0.009 | 1.36 | (0.29, | 2.43) | 0.013 |
| South Asian covariate | 0.39 | (-0.63 | 1.41) | 0.452 | 0.83 | (-0.31 | 1.97) | 0.152 | 0.75 | (-0.37 | 1.88) | 0.190 |

p/min, per minute; p/hr, per hour
